# A new fast method for inferring multiple consensus trees using *k*-medoids

**DOI:** 10.1186/s12862-018-1163-8

**Published:** 2018-04-05

**Authors:** Nadia Tahiri, Matthieu Willems, Vladimir Makarenkov

**Affiliations:** Département d’Informatique, Université du Québec à Montréal, Case postale 8888, Succursale Centre-ville, Montréal, H3C 3P8 Canada

**Keywords:** Cluster validity index, Consensus tree, *k*-medoids, Phylogenetic tree, Robinson and Foulds topological distance

## Abstract

**Background:**

Gene trees carry important information about specific evolutionary patterns which characterize the evolution of the corresponding gene families. However, a reliable species consensus tree cannot be inferred from a multiple sequence alignment of a single gene family or from the concatenation of alignments corresponding to gene families having different evolutionary histories. These evolutionary histories can be quite different due to horizontal transfer events or to ancient gene duplications which cause the emergence of paralogs within a genome. Many methods have been proposed to infer a single consensus tree from a collection of gene trees. Still, the application of these tree merging methods can lead to the loss of specific evolutionary patterns which characterize some gene families or some groups of gene families. Thus, the problem of inferring multiple consensus trees from a given set of gene trees becomes relevant.

**Results:**

We describe a new fast method for inferring multiple consensus trees from a given set of phylogenetic trees (i.e. additive trees or *X*-trees) defined on the same set of species (i.e. objects or taxa). The traditional consensus approach yields a single consensus tree. We use the popular *k*-medoids partitioning algorithm to divide a given set of trees into several clusters of trees. We propose novel versions of the well-known Silhouette and Caliński-Harabasz cluster validity indices that are adapted for tree clustering with *k*-medoids. The efficiency of the new method was assessed using both synthetic and real data, such as a well-known phylogenetic dataset consisting of 47 gene trees inferred for 14 archaeal organisms.

**Conclusions:**

The method described here allows inference of multiple consensus trees from a given set of gene trees. It can be used to identify groups of gene trees having similar intragroup and different intergroup evolutionary histories. The main advantage of our method is that it is much faster than the existing tree clustering approaches, while providing similar or better clustering results in most cases. This makes it particularly well suited for the analysis of large genomic and phylogenetic datasets.

## Background

Various methods for computing a consensus tree for a given set of phylogenetic trees have been proposed [1]. The most known types of consensus trees are the strict consensus tree, the majority consensus tree and the extended majority consensus tree [1, 2]. The strict consensus tree contains only the edges that are common to all input trees. The majority consensus tree contains the edges that are present in more than 50% of the input trees, although higher percentages may also be considered. According to the extended majority rule, the consensus tree includes all of the majority edges to which compatible residual edges are added gradually, starting with the most frequent ones. Extended majority consensus trees are the most frequently used consensus trees in evolutionary biology because they are usually much better resolved (i.e. have lower mean degree of internal nodes) than strict and majority consensus trees [2].

The output of most conventional consensus tree algorithms is a single consensus tree [1]. However, in many practical situations it is much more appropriate to infer several consensus trees. In biology, it is often risky to group phylogenetic trees corresponding to different sets of genes. Each gene has its own evolutionary history which can substantially differ from evolutionary histories of other genes. For example, some individual genes or gene clusters (e.g. operons) affected by specific horizontal gene transfer events will display different evolutionary patterns than the rest of genes under study [3–8]. The evolutionary history of such genes or gene clusters will be depicted by phylogenetic trees having different topologies from that of the species tree which represents the evolution of genes that did not undergo gene transfers. Furthermore, the homogeneity of a given set of genes can be also affected by ancient duplication events causing the emergence of paralogous alleles.

There are several computational tools for analyzing and visualizing sets of incompatible phylogenetic trees, including SplitsTree [9], Dendroscope [10] and DensiTree [11]. These programs allow for inferring different kinds of phylogenetic networks which can be viewed as alternatives to multiple consensus trees. Holland et al. [12] were among the first to discuss a consensus building approach using splits network. Holland et al. compared gene trees of yeast genomes and demonstrated that consensus networks can be useful to depict hidden contradictory signals existing in species phylogenies.

**Fig. 1:**
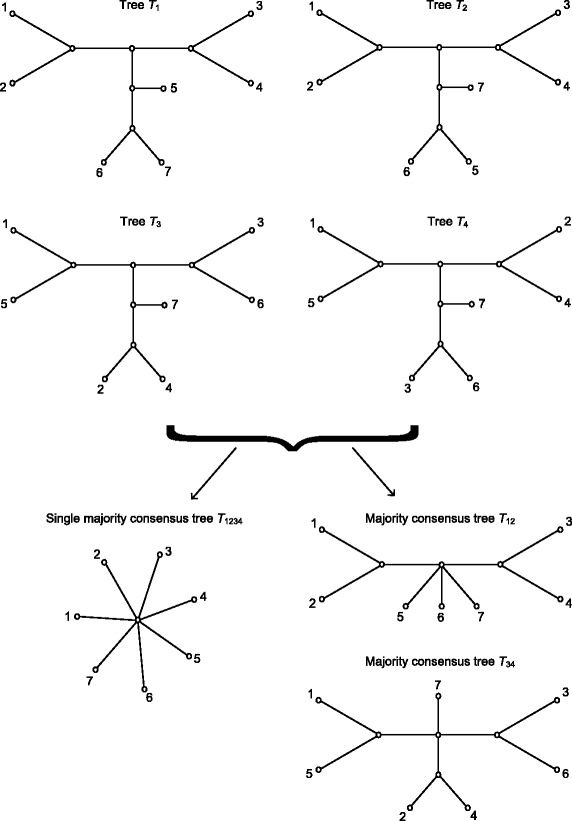
Four phylogenetic trees *T*_1_, *T*_2_, *T*_3_ and *T*_4_ defined on the same set of seven leaves. Their majority-rule consensus tree is a star tree *T*_1234_. The majority-rule consensus trees, *T*_12_ and *T*_34_, constructed for the pairs of topologically close trees: *T*_1_ and *T*_2_, and *T*_3_ and *T*_4_, respectively

Thus, the question whether a unique consensus tree or multiple consensus trees best characterize a given set of phylogenies arises as an alternative to phylogenetic network reconstruction approaches. If the given phylogenies are topologically congruent, they should be combined into a single consensus tree. However, if these phylogenies encompass conflicting genetic signals, they should be organized into multiple consensus trees, each of which accounts for a specific evolutionary pattern [13–15]. Figure 1 shows four phylogenetic trees *T*_1_, *T*_2_, *T*_3_ and *T*_4_ with seven leaves. Here, the solution consisting of two majority-rule consensus trees, *T*_12_ and *T*_34_, seems to be much more appropriate than the solution consisting of a single majority consensus tree, *T*_1234_, i.e. a star tree here, given by the traditional majority consensus approach.

In this paper we describe a new algorithm for determining clusters of homogeneous trees which can be combined in order to infer multiple consensus trees. The idea of building multiple consensus trees was originally formulated by Maddison [16] who found that consensus trees of some subsets of a given set of trees may differ and that they are usually better resolved than the consensus tree of the whole set. Then, Stockham et al. [17] proposed two variants of a tree clustering algorithm based on *k*-means, which were meant to infer a set of strict consensus trees (called characteristic trees) minimizing the information loss. However, these methods were very expensive in terms of the running time because the consensus trees had to be determined for each set of clusters in all intermediate partitioning solutions tested by *k*-means. Bonnard et al. [13] described a method, called Multipolar Consensus, to display all the splits of a given set of phylogenetic trees having a support above a predefined threshold, using a minimum possible number of consensus trees. The authors indicated that biologically relevant secondary signals, which would be normally absent in a classical consensus tree, can be captured by the Multipolar Consensus method thus providing a convenient exploratory tool for phylogenetic analysis. This method allows one to display more secondary evolutionary signals than it is proposed by the extended majority rule consensus without making possible arbitrary choices which are usually made in this consensus method. In his recent paper, Guénoche [14] has presented a method for partitioning phylogenetic trees into one cluster (*K*=1, when given gene trees are homogeneous) or several clusters (*K*>1, when given gene trees are divergent). A generalized partition score, computed over a set of tree partitions, is calculated by the Guénoche method in order to determine the number of clusters, *K*, in which a given set of gene trees should be partitioned. Guénoche validated his method on both simulated data, i.e. random sets of trees organized in different topological groups, and real data, i.e. a set of non homogeneous gene trees of 30 *E*.*coli* strains assumed to be affected by horizontal gene transfers. The MCT (Multiple Consensus Trees) program developed by the author remains one of the rare pieces of software for inferring multiple consensus trees, available for the research community.

We will describe a new tree clustering method that relies on specific versions of the Silhouette (*SH*) [18] and Caliński-Harabasz (*CH*) [19] indices adapted for tree clustering with *k*-medoids. These cluster validity indices will be used to determine the best partitioning obtained over multiple random starts of *k*-medoids [20] when the number of clusters is fixed and then to select the optimal number of clusters for a given set of trees.

## Methods

### *K*-medoids algorithm adapted for tree clustering

A phylogenetic tree is an unrooted leaf-labeled tree in which each internal node, representing an ancestor of contemporary species, has at least two children and all leaves, representing contemporary species, have different labels [2, 21, 22]. Our algorithm takes as input a set of phylogenetic trees Π defined on the same set of leaves and returns as output one or several consensus trees. Each consensus tree represents a subset (i.e. group, class or cluster) of trees from Π. For each cluster identified, the algorithm returns a list of its elements (i.e. phylogenetic trees) and the corresponding consensus tree. The output is also accompanied by some statistics (e.g. the value of the selected cluster validity index). Our method uses a version of the popular *k*-medoids algorithm suitable for tree clustering. The *k*-medoids algorithm [20] is a clustering method which can be viewed as a robust version of the popular *k*-means algorithm. The *k*-medoids algorithm divides *N* elements (i.e. phylogenetic trees in our case) into *K* clusters using the cluster centers (i.e. the medoids) which belong to the set of original elements (i.e. original trees from Π in our case). The medoid of a given cluster is chosen to minimize the overall distance to the other elements of this cluster. The content of each cluster is chosen to minimize the total intracluster distance. Generally, the most commonly used distances in the framework of *k*-medoids are the Euclidean distance, the Manhattan distance and the Minkowski distance.

Several measures have been proposed to estimate the distance between phylogenetic trees. The most popular of them are the Robinson and Foulds (*RF*) topological distance [23], the quartet distance [24], the SPR (Subtree Prune and Regraft) distance [25], the MAST (Maximum Agreement Subtree) distance [26] and the bipartition dissimilarity [3]. Unfortunately, the popular SPR distance, which is often used to identify horizontal gene transfer events, takes an exponential time to calculate. This is also the case of the MAST distance which cannot be calculated in polynomial time. The *RF* and the quartet distances are topological distances which are among the quickest to calculate. Indeed, both of them can be computed in $\mathcal {O}(n^{2})$ when two Newick strings, representing two phylogenies with *n* leaves defined on the same set of species, are considered. Moreover, Barthélemy and McMorris [27] have shown that the majority consensus tree of a set of trees is a median tree of this set in the sense of the *RF* distance [27]. Thus, the *RF* topological distance seems to be an appropriate distance to be used within *k*-means or *k*-medoids algorithms adapted for phylogenetic tree clustering. In our work, we define the median tree as a tree based on the *RF* distance. It is worth noting that one could also use an alternative type of median trees, those based on the SPR distance [28], to infer SPR-distance-based multiple consensus trees. For instance, Bruen and Bryant showed that the maximum parsimony tree can be viewed as a type of median consensus tree in the sense of the SPR distance.

The Robinson and Foulds distance [14, 23, 29, 30] between two trees is a well-known distance used in computational biology to compare the topologies of two phylogenetic trees defined on the same set of species. The Robinson and Foulds distance is a topological distance. It does not take into account the lengths of the tree edges.

The time complexity of a typical implementation of the *k*-medoids algorithm is $ \mathcal {O}\left (K \times (N - K)^{2} \times i \times M \right)$, where *K* is the number of clusters, *i* is the number of iterations in *k*-medoids and *M* is the number of variables characterizing each of the *N* objects. One of the advantages of our new algorithm based on the RF distance is that it does not need to recompute the consensus trees for intermediate clusters of trees. Instead, it estimates the quality of each intermediate clustering using an approximate formula. This allows a much faster partitioning of a given set of phylogenetic trees into *K* clusters without losing the quality of the obtained consensus trees.

In the case of tree clustering using *k*-means, the objective function to be minimized can be defined as follows:

1 $$ OF = \sum^{K}_{k=1}\sum^{N_{k}}_{i=1} RF\left(T^{maj}_{k},T_{ki}\right),  $$

where *K* is the number of clusters, *N*_*k*_ is the number of trees in cluster *k*, *RF* is the Robinson and Foulds topological distance between two phylogenetic trees with *n* leaves, *T*_*ki*_ is the tree *i* of cluster *k* and $T^{maj}_{k}$ is the majority rule consensus tree of cluster *k*. Still, the computation of the majority rule consensus tree or of the extended majority rule consensus tree is time-consuming. The time complexity of the method computing the majority or the extended majority rule consensus tree is $ \mathcal {O}\left (n^{2} + nN^{2}\right)$ [2], where *N* is the number of trees and *n* is the number of leaves in each tree. Jansson et al. [31] have recently proposed a number of deterministic algorithms for constructing the majority rule consensus tree and some of its variants. The authors presented the algorithms running in $\mathcal {O}\left (nN \times log N \right)$ time - for a majority rule consensus tree, in $\mathcal {O}(nN)$ time - for a loose consensus tree, and in $\mathcal {O}\left (n^{2}N\right)$ time - for a greedy consensus tree. However, if the trees are defined by their Newick strings, as it is typically done in evolutionary biology [32], one will need a conversion program to transform each Newick string into the format required by the algorithms of Jansson et al.

Stockham et al. [17] proposed two variants of the popular *k*-means algorithm to infer a set of strict consensus trees (called characteristic trees) that minimize the loss information. However, the approach of Stockham et al. seems to be very expensive in terms of the running time because in their approach the consensus trees are determined for each set of clusters in all intermediate partitioning solutions tested by *k*-means. We present a new algorithm that does not need to recompute the consensus trees at each of its iterations. The time complexity of a straightforward tree partitioning algorithm, such as the algorithm of Stockham et al. [17] based on *k*-means, which recomputes the consensus trees after each basic *k*-means operation consisting of relocating an object (i.e. tree) from one cluster to another and then in reassessing the value of the objective function (Formula 1), is $ \mathcal {O}\left (K \times n \times \left (n + N^{2}\right)\times i\right)$.

We propose to use the following approximate formula, based on the properties of *k*-medoids:

2 $$ OF_{med} = \sum^{K}_{k=1}\sum^{N_{k}}_{i=1} RF\left(T^{m}_{k},T_{ki}\right),  $$

where $T^{m}_{k}$ is the medoid tree of cluster *k*. The medoid tree $T^{m}_{k}$ of cluster *k* is the tree belonging to cluster *k* that minimizes the sum of *RF* distances between it and all other trees in *k*. By contrast with the *k*-means-based approach, we do not need to compute in our algorithm cluster centroids or majority consensus trees of clusters. Using Formula 2, we reduce the time complexity of the method to $ \mathcal {O}\left (nN^{2} + K \times (N - K)^{2} \times i\right)$, where $ \mathcal {O}\left (nN^{2}\right)$ is the time complexity of precalculating the matrix of pairwise *RF* distances of size (*N*×*N*) between all trees in Π.

### Silhouette cluster validity index adapted for tree clustering with *k*-medoids

The first cluster validity index we consider in this study is the Silhouette width (*SH*) [18]. This index assesses the average rate of similarity between the objects belonging to the same cluster (i.e. a cohesion function) versus the rate of similarity between the objects of different clusters (i.e. a separation function). The Silhouette width for cluster *k* is defined as follows:

3 $$ sh(k) = \frac{1}{N_{k}}\left[ \sum^{N_{k}}_{i=1}\frac{b(i)-a(i)}{\max(a(i),b(i))}\right],  $$

where *N*_*k*_ is the number of elements (i.e. trees) in cluster *k*, *a*(*i*) is the average distance between the element *i* and all other elements of *k* and *b*(*i*) is the smallest of all distances between the element *i* of cluster *k* and the elements in the other clusters (i.e. those different from *k*). The optimal number of clusters corresponds to the highest value of Silhouette.

The following equations for calculating *a*(*i*) and *b*(*i*) can be used when clustering trees. The formula for *a*(*i*) is as follows:

4 $$ a(i) = \frac{\sum_{j=1}^{N_{k}} RF\left(T_{ki},T_{kj}\right)}{N_{k}},  $$

where *T*_*ki*_ is the tree *i* of cluster *k* and *T*_*kj*_ is the tree *j* of cluster *k*. The formula for *b*(*i*) is as follows:

5 $$ b(i) = \min\limits_{1 \leq k^{\prime} \leq K, \mathrm{\:and\:} k^{\prime} \ne k}\frac{\sum_{j=1}^{N_{k^{\prime}}}RF\left(T_{ki},T_{k^{\prime}j}\right)}{N_{k^{\prime}}},  $$

where $T_{k^{\prime }j}$ is the tree *j* of cluster *k*^′^ and $N_{k^{\prime }}$ is the number of trees in cluster *k*^′^.

Finally, the optimal number of clusters *K* corresponds to the maximum average Silhouette width, *SH*(*K*), defined as follows:

6 $$ SH(K)=\sum_{k=1}^{K}\left[ sh(k) \right]/K.  $$

### Caliński-Harabasz cluster validity index adapted for tree clustering with *k*-medoids

The second cluster validity index we consider here is the Caliński-Harabasz index (*CH*) [19]. This criterion is a ratio between the overall between-cluster distance and the overall within-cluster distance, calculated by taking into account the number of degrees of freedom. The exact formula for the *CH* computation is as follows:

7 $$ CH = \frac{SS_{B}}{SS_{W}} \times \frac{N-K}{K-1},  $$

where *SS*_*W*_ is the overall within-cluster distance involving the elements of the same cluster, *SS*_*B*_ is the overall between-cluster distance involving the elements of different clusters, *K* is the number of clusters and *N* is the number of elements. The optimal number of clusters corresponds to the maximum of *CH*.

The traditional formula for calculating the overall within-cluster variance, *SS*_*W*_, is as follows:

8 $$ SS_{W} = \sum^{K}_{k=1}\sum^{}_{y \in C_{k}}\|y-m_{k}\|^{2},  $$

where *y* is an element of cluster *C*_*k*_, *m*_*k*_ is the centroid of cluster *k* and ∥*y*−*m*_*k*_∥^2^ is the Euclidean distance (*L*^2^ norm) between *y* and *m*_*k*_.

The traditional formula for calculating the overall between-cluster variance, *SS*_*B*_, is as follows:

9 $$ SS_{B} = \sum^{K}_{k=1} N_{k} \|m_{k}-m\|^{2},  $$

where *N*_*k*_ is the number of elements in cluster *k*, *m* is the overall mean of the sample data and ∥*m*_*k*_−*m*∥^2^ is the Euclidean distance between *m*_*k*_ and *m*. Good clusterings have a large overall between-cluster distance (*SS*_*B*_) and a small overall within-cluster distance (*SS*_*W*_).

Clearly, we cannot use the Euclidean distance when clustering trees [33]. The Robinson and Foulds topological distance (*RF*) has been used instead. Namely, the following formulas for the overall within-cluster and between-cluster distances were used in our study:

10 $$ SS_{W} = \sum^{K}_{k=1} \sum^{N_{k}}_{i=1} RF\left(T^{m}_{k},T_{ki}\right), \text{and}  $$

11 $$ SS_{B} = \sum^{K}_{k=1} N_{k} \times RF\left(T^{m}_{k},T^{m}\right),  $$

where $T^{m}_{k}$ is the medoid tree of cluster *k*, *T*_*ki*_ is the tree *i* of cluster *k* and *T*^*m*^ is the medoid tree of the sample data.

### Adjusted Rand index

The quality of clustering results was evaluated by using the Adjusted Rand Index (*ARI*) [34–36]. The values of *ARI* are located in the interval [-1; 1]. When two partitions are exactly the same, the corresponding value of *ARI* is 1. This popular index is the corrected for chance version of the Rand index [37, 38]. *ARI* is often used in simulations to compare the known original partitions with those generated by methods under study.

Given a set of *n* objects and two partitions of these objects, namely *X*=*X*_1_,*X*_2_,…,*X*_*r*_ with *r* clusters and *Y*=*Y*_1_,*Y*_2_,…,*Y*_*s*_ with *s* clusters, the overlap between *X* and *Y* can be summarized using a contingency matrix [*n*_*ij*_], where each entry *n*_*ij*_ denotes the number of objects in common between partitions *X*_*i*_ and *Y*_*j*_.

The ARI index is calculated using Formula (12):

12 $$ {}ARI = \frac{\sum_{ij}{{n_{ij} \choose 2}-\left[ \sum_{i}{{a_{i} \choose 2}} \sum_{j}{{b_{j} \choose 2}} \right]}\mathbin{/}{{n \choose 2}}}{\frac{1}{2}\left[ \sum_{i}{{a_{i} \choose 2}} + \sum_{j} {{b_{j} \choose 2}} \right] - \left[ \sum_{i}{{a_{i} \choose 2}} \sum_{j} {{b_{j} \choose 2}} \right]\mathbin{/}{{n \choose 2}}},  $$

where *n*_*ij*_=|*X*_*i*_∩*Y*_*j*_|, $a_{i} = \sum ^{s}_{j=1}|X_{i} \cap Y_{j}|$ and $b_{j} = \sum ^{r}_{i=1}|X_{i} \cap Y_{j}|$, and *X*_*i*_ and *Y*_*j*_ are the sets of objects in clusters *i* and *j*, respectively.

## Results

### Simulation design

We tested our new algorithm for computing multiple consensus trees using the two following simulation protocols.

**Fig. 2:**
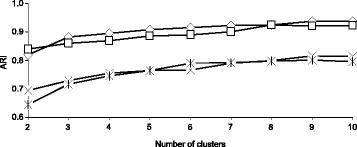
Classification performances of the four versions of our *k*-medoids tree clustering algorithm in terms of *ARI* with respect to the number of clusters, ranging from 2 to 10. The four tested versions of our algorithm were those based on: 1) *SH* with *RF* (*◇*), 2) *CH* with *RF* (×), 3) *SH* with *RF* squared (*□*) and 4) *CH* with *RF* squared (). The coalescence rate parameter in the HybridSim program was fixed to 5 in this simulation. The presented results are the averages taken over all considered numbers of tree leaves

**Fig. 3:**
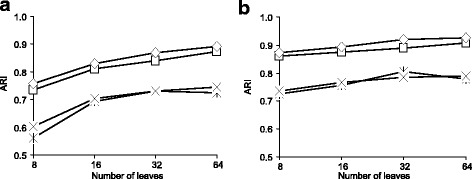
Classification performances of the four versions of our *k*-medoids tree clustering algorithm in terms of *ARI* with respect to the number of tree leaves: **a** the case of 2 to 5 clusters and **b** the case of 6 to 10 clusters. The four tested versions of our algorithm were based on: 1) *SH* with *RF* (*◇*), 2) *CH* with *RF* (×), 3) *SH* with *RF* squared (*□*) and 4) *CH* with *RF* squared (). The coalescence rate parameter in the HybridSim program was fixed to 5 in this simulation. The presented results are the averages taken over all considered numbers of clusters

**Fig. 4:**
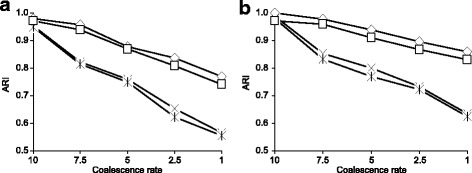
Classification performances of the four tested versions of our *k*-medoids tree clustering algorithm in terms of *ARI* with respect to the coalescence rate: **a** the case of 2 to 5 clusters and **b** the case of 6 to 10 clusters. The four tested versions of our algorithm were based on: 1) *SH* with *RF* (*◇*), 2) *CH* with *RF* (×), 3) *SH* with *RF* squared (*□*) and 4) *CH* with *RF* squared (). The coalescence rate parameter in the HybridSim program varied from 10 to 1 in this simulation. The presented results are the averages taken over all considered numbers of clusters and all considered numbers of tree leaves

Our first simulation included two main steps. During the first step, we randomly generated a species phylogenetic tree (i.e. first consensus tree here) *T*_1_ with *n* leaves using the HybridSim [39] program. Then, using the same program, we generated *K*−1 other consensus trees, *T*_2_, …, *T*_*K*_ with *n* leaves, each of which differed from *T*_1_ by a specified number of hybridization events (the value of the *hybridizationrate* parameter in the HybridSim program varied from 1 to 4 in our simulation; it was drawn randomly using a uniform distribution). In our first simulation, the number of clusters, *K*, ranged from 2 to 10 (see Figs. 2, 3 and 4), while the number of tree leaves, *n*, was taking the values 8, 16, 32 and 64 (see Fig. 3).

The HybridSim program developed by Woodhams et al. [39] allows generation of phylogenies in the presence of hybridization and horizontal gene transfer events. This program can generate trees differing from each other by a specified number of coalescence/incomplete lineage sorting producing patterns of incongruence across gene trees. In our simulations with HybridSim, we varied the values of the *hybridizationrate* (as indicated above) and the *coalescencerate* (as indicated below) parameters. The rest of parameters used in our simulations were the default parameters of HybridSim.

During the second step of the first simulation, for each consensus phylogenetic tree *T*_*i*_ (*i* = 1, …, *K*) representing cluster *i*, we generated a set of 100 trees belonging to cluster *i* using a specified value of the coalescence rate parameter in HybridSim. In our study, the value of this coalescence parameter, introducing noise into gene phylogenies, varied between 10 (low noise) and 1 (high noise). Thus, each element *T* of cluster *i* differed from the consensus tree *T*_*i*_ of this cluster by a certain (fixed) coalescence degree. The simulation results presented in Figs. 2 and 3 correspond to the case in which the coalescence rate parameter in HybridSim was fixed to 5. Figure 4 illustrates how the methods’ results change with respect to the change in the coalescence parameter. The number of clusters, *K*, was assumed to be known in this simulation. The strategies based on both the squared and non-squared *RF* distances (used in Formula 2) were evaluated.

**Fig. 5:**
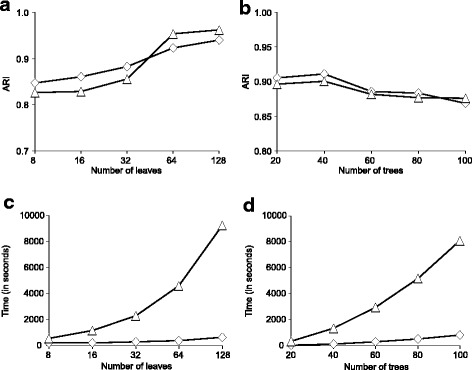
Comparison of our algorithm (*◇*) based on the *k*-medoids clustering, the non-squared *RF* distance and the *SH* cluster validity index to the traditional approach (△) based on the *k*-means clustering, on the squared *RF* distance and on the recomputing the majority consensus trees within *k*-means (Stockham et al. [17]). The coalescence rate parameter in the HybridSim program was fixed to 5 in this simulation. The comparison was made in terms of *ARI* (panels **a** and **b**) and the running time (measured in seconds) of the methods (panels **c** and **d**) with respect to the number of tree leaves and trees

In the second simulation, we compared our algorithm based on the Silhouette index and the non-squared Robinson and Foulds distance (Formula 2) with the traditional approach based on the recalculation of majority-rule consensus trees, representing the cluster centroids, after each basic operation of *k*-means (a variant of Formula 1 using the squared *RF* distance, as suggested by Stockham et al. [17]). This comparison was performed in terms of quality of clustering results returned by competing methods (Fig. 5a and b) and of running time (Fig. 5c and d). The number of tree leaves, *n*, in this second simulation was equal to: 8, 16, 32, 64 and 128. The number of clusters, *K*, in the second simulation was equal to 5 and the coalescence rate parameter in the HybridSim program was fixed to 5 in this simulation. Once again, 100 different datasets were generated for each parameter combination and the number of clusters was assumed to be known. Our simulations were carried out using a 64-bit computer equipped with an Intel i5-4690T CPU (2.5 GHz) and 8 Gb of RAM.

The results of the first simulation are illustrated in Figs. 2, 3 and 4. The presented results are the averages taken over all combinations of our parameters (see the “Simulation study” section), except the featured one (i.e. the number of clusters in Fig. 2, the number of tree leaves in Fig. 3 and the coalescence rate in Fig. 4). The obtained results are discussed later on in the “Discussion” section.

### Clustering trees of 47 ribosomal proteins of Archaea

**Fig. 6:**
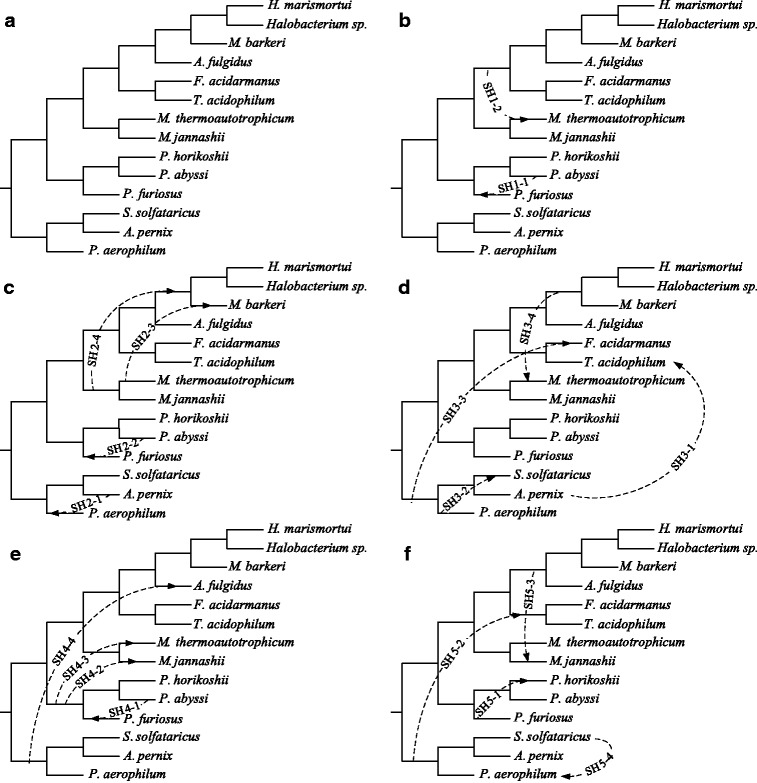
**a** Species tree for the Archaea dataset and *five consensus* horizontal gene transfer scenarios (panels **b** to **f**) obtained for 47 protein trees, originally studied by Matte-Tailliez et al. [4], using the *SH* cluster validity index and the non-squared *RF* distance in the *k*-medoids tree clustering algorithm

We applied the new algorithm to analyze the evolution of 47 ribosomal proteins of 14 organisms of Archaea, including 11 species of Euryarchaeota and 3 species of Crenarchaeota. These data were originally studied by Matte-Tailliez et al. (see Fig. 1a in [4] or Fig. 6a in our paper). Matte-Tailliez et al. inferred a single species phylogenetic tree after the concatenation of the considered protein sequences (see Fig. 6a). However, the evolution of each of these proteins can be represented by its own phylogenetic tree. The cluster analysis of these trees can tell us how many different evolutionary scenarios characterize the evolution of these sequences (i.e. how many clusters of trees exist in the protein tree dataset). We first considered the complete set of 52 multiple sequence alignments of archaeal ribosomal proteins studied by Matte-Tailliez et al. We selected for our analysis 47 of these 52 alignments, i.e. those including the data for the same 14 archaeal organisms. The 5 remaining alignments were incomplete (i.e. they included 12 or 13 organisms only). Using the 47 complete alignments, we inferred 47 phylogenetic trees by means of the PHYML method [40] (these alignments and trees are available at: https://github.com/TahiriNadia/CKMedoidsTreeClustering/).

First, we carried out the version of our *k*-medoids tree clustering algorithm based on the *SH* index (Formulas 3 to 6) and the non-squared *RF* distance in order to infer a partitioning of the obtained set of 47 trees. The maximum of *SH* was attained with five clusters (*K*=5) which correspond to five different horizontal gene transfer scenarios presented in Fig. 6 (panels b to f).

The first cluster contained 11 trees, the second 4 trees, the third 20 trees, the fourth 11 trees and the fifth 1 tree. We inferred the extended majority consensus trees, *SH*1, *SH*2, *SH*3, *SH*4 and *SH*5, for these five clusters of trees. Afterwards, using the gene transfer detection algorithm by Boc et al. [3], we identified the scenarios of horizontal gene transfer events which reconcile the species tree (Fig. 6a) and each of the obtained consensus trees, *SH*1 to *SH*5. In the end of the tree reconciliation process, consisting of SPR moves (corresponding to horizontal gene transfers) of clusters of the species tree, the transformed topology of the species tree becomes identical to that of the gene tree. The version of the algorithm available on the T-Rex web site [41] was used in our computations.

**Fig. 7:**
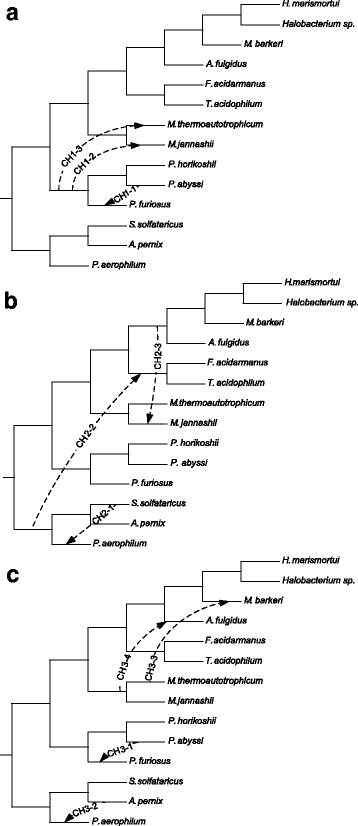
*Three consensus* horizontal gene transfer scenarios (panels **a** to **c**) obtained for 47 protein trees, originally studied by Matte-Tailliez et al. [4], using the *CH* cluster validity index and the non-squared *RF* distance in the *k*-medoids tree clustering algorithm

Second, we carried out the version of our *k*-medoids tree clustering algorithm based on the *CH* index (Formulas 7 to 11) and the non-squared *RF* distance to classify the same set of 47 gene trees. The maximum of *CH* was attained with three clusters (*K*=3). Here, the first cluster contained 25 trees, the second 14 trees and the third 8 trees. We then inferred the extended majority consensus trees, *CH*1, *CH*2 and *CH*3, for these clusters of trees. Similarly to the case of *SH*, we identified the scenarios of horizontal gene transfer events that reconcile the species tree (Fig. 6a) and each of the consensus trees *CH*1, *CH*2 and *CH*3 (see Fig. 7, panels a to c).

## Discussion

### Simulation study

The curves depicted in Fig. 2 indicate that the clustering quality provided by our algorithm depends on the number of clusters, the cluster validity index (*SH* or *CH*) and the selected objective function (i.e. the non-squared *RF* distance or the squared *RF* distance in Formula 2). Obviously, we were not able to address the case of homogeneous data (i.e. *K* = 1) in this study because the *SH* and *CH* cluster validity indices are not adapted for the case of one cluster only. The *ARI* results improve noticeably when the number of clusters, *K*, increases from 2 to 6 and stabilize starting from 6 clusters. Also, the strategy based on the *SH* cluster validity index and the non-squared *RF* distance outperforms the three other competing strategies regardless of the number of clusters.

Figure 3 shows a slight increase in the *ARI* values as the number of tree leaves increases. Once again, the scores of *ARI* for the *SH* criterion are higher than those for the *CH* criterion and the methods based on the non-squared *RF* distance are slightly more efficient than those based on the squared *RF* metric. One can also observe that the *ARI* scores become more stable when the number of clusters varies between 6 and 10 (Fig. 3b) compared to the case when it varies between 2 and 5 (Fig. 3a).

Figure 4 shows that the *CH* index is more affected by coalescence (i.e. noise) than *SH* when clustering trees using *k*-medoids. Once again, one can notice that both versions of our algorithm, based on *SH* and *CH*, yield better results when the non-squared *RF* distance is used in Formula 2 instead of its squared counterpart. It is worth noting that Stockham et al. [17] used the squared *RF* distance in their algorithm. Given these results, our second simulation (see Fig. 5) was conducted with the algorithm based on the Silhouette index and the non-squared Robinson and Foulds distance.

The results of the second simulation are shown in Fig. 5. The curves presented in Fig. 5 (a and b) indicate that the new algorithm based on Formulas 2 to 6 works better than the straightforward tree clustering approach by Stockham et al. [17] in terms of the clustering quality when the number of tree leaves varies from 8 to 32, but is slightly less efficient than the approach by Stockham et al. when the number of tree leaves varies from 64 to 128. However, our algorithm is by far the best method in terms of the running time for both simulation parameters considered: the number of tree leaves (Fig. 5c) and the number of trees (Fig. 5d). These results suggest that our new algorithm is well suited for the analysis of large phylogenetic datasets.

### Analysis of clustering results obtained for 47 trees of ribosomal proteins of Archaea

Horizontal gene transfer scenarios found using the SH index account for five different histories which characterize the evolution of the 47 ribosomal proteins considered. Two transfers predicted for these data by Boc et al. (see Fig. 6 in [3]), which are in agreement with the results of Matte-Tailliez et al. [4] and Boc et al. 2013 [42], are present in these five scenarios. Precisely, the transfers - *SH*3−2 (or its equivalent transfer *SH*5−4) and *SH*5−1 - have been predicted by Boc et al. 2010 (see Fig. 6 in [3]), the transfers - *SH*3−2 (or its equivalent transfer *SH*5−4) and *SH*3−1 - have been predicted by Boc et al. 2013 (see Fig. 2b in [42]). Finally, the transfers - *SH*1−1 (or its equivalent transfers *SH*2−2 and *SH*4−1), *SH*5−1, *SH*3−2 (or its equivalent transfer *SH*5−4), *SH*2−1, *SH*4−3 and *SH*4−2 - have been predicted by Boc et al. 2013 (see Fig. 3 in [42]) as partial horizontal gene transfers (i.e. transfers leading to the formation of chimeric genes composed of portions of two or more coding sequences; see [43]).

We also used the MCT program by Guénoche [14] (Multiple Consensus Trees) to analyze this Archaea dataset. The average linkage hierarchical algorithm and the Robinson and Foulds distance were the parameters which we selected in MCT. We compared our consensus trees of classes with the consensus trees found by the algorithm by Guénoche[14]. For example for *K*=5 (this was the optimal number of clusters found using our algorithm with the *SH* index), the MCT program returned consensus gene trees whose topologies led to the horizontal gene transfers *SH*2−1, *SH*2−3, *SH*4−2 and *SH*4−3 (see Fig. 6).

Horizontal gene transfer scenarios found using the CH index account for three different evolutionary histories of the 47 ribosomal proteins under examination. Here, the transfer - *CH*2−1 - has been predicted by Boc et al. 2010 (see Fig. 6 in [3]), the transfer - *CH*2−2 - has been predicted by Boc et al. 2013 (see Fig. 2b in [42]), and finally the transfers - *CH*1−1 (or its equivalent transfer *CH*3−1), *CH*1−2, *CH*1−3, *CH*2−1, *CH*3−2 and *CH*3−4 - have been predicted by Boc et al. 2013 as partial horizontal gene transfer events (see Fig. 3 in [42]). Interestingly, all the transfers found in the horizontal gene transfer scenarios shown in Fig. 7 (a-c) can be found in the gene transfer scenarios presented in Fig. 6 (b-f).

Finally, we ran the MCT program with *K*=3 (this was the optimal number of clusters found using our algorithm with the *CH* index) and compared the obtained consensus trees with those found by our method. The consensus trees found by MCT in this case allowed for four horizontal gene transfers which were equivalent to the transfers *CH*1−2, *CH*1−3, *CH*3−2 and *CH*3−3 (see Fig. 7) found by our algorithm with the *CH* index.

Still using the horizontal gene transfer detection algorithm by Boc et al. [3], we found scenarios of gene transfer events reconciling the species tree (Fig. 7a) and the obtained consensus trees which play the role of gene trees in this context. The overall horizontal gene transfer results comparing the frequencies of the intragroup and intergroup gene transfers found by our algorithm using the *SH* and *CH* indices are reported Table 1. They suggest that gene transfers have been more frequent within the species of the same phylum than between the species of different phyla (i.e. Crenarchaeota and Euryarchaeota).

**Table 1:** Gene transfer statistics for 47 ribosomal protein trees constructed for 14 species of Archaea obtained using the *SH* and *CH* cluster validity indices and the non-squared *RF* distance in the *k*-medoids tree clustering algorithm

Criterion	Type of gene transfer	Number of transfers detected	Percentage of transfers detected
SH	Intragroup	14	77.78%
	Intergroup	4	22.22%
CH	Intragroup	9	90%
	Intergroup	1	10%

The Crenarchaea group is composed of *S. solfactaricus*, *A. pernix* and *P. aerophilum* species, and the Euryarchaeota group is composed of *P. furiosus*, *P. abyssi*, *P. horikoshii*, *M. jannashii*, *M. thermoautotrophicum*, *T. acidophilum*, *F. acidamanus*, *A. fulgidus*, *M. barkeri*, *Halobacterium sp.* and *H. marismortui* species

## Conclusions

In this article we described a new algorithm for partitioning a set of phylogenetic trees into several clusters in order to infer multiple consensus trees. We presented new formulas allowing for using the popular Silhouette and Caliński-Harabasz cluster validity indices as well as the Robinson and Foulds topological distance in the framework of tree clustering based on the popular *k*-medoids algorithm. The new algorithm can be used to address a number of important issues in evolutionary biology, such as the identification of genes having similar evolutionary histories, e.g. those that have undergone the same horizontal gene transfers or those that have been affected by the same ancient duplication events. The presented algorithm could be extended to the case where the input trees have different, but mutually overlapping, sets of leaves. In order to compute the Robinson and Foulds topological distance between such trees, we could first reduce them to the common set(s) of leaves. After this reduction, the Robinson and Foulds distance normalized by its maximum value, which is 2*n*−6 for two binary trees with *n* leaves, could be used in Formulas 1 and 2 in order to infer multiple consensus trees. Overall, good performances achieved by the new algorithm in terms of both clustering quality and running time makes it well suited for the analysis of large genomic and phylogenetic datasets. A C++ program, called KMTC (*K*-Medoids Tree Clustering), implementing the discussed tree partitioning algorithm is freely available at: https://github.com/TahiriNadia/CKMedoidsTreeClustering/.

## Abbreviations

ARI: Adjusted Rand index
CH: Caliń
ski-Harabasz cluster validity indice; KMTC: *K*-Medoids tree clustering
MCT: Multiple consensus trees program
RF: Robinson and Foulds topological distance
SH: Silhouette cluster validity indice
SS_B_: Overall between-cluster distance
SS_W_: Overall within-cluster distance
ToL: Tree of life project
